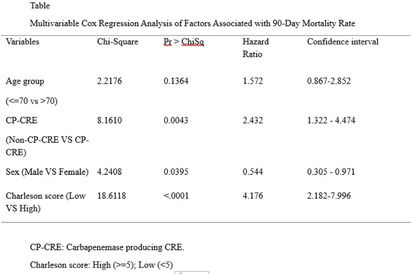# Survival Analysis of Carbapenem Resistant Enterobacterales (CRE) Cases in Davidson and Surrounding Counties, Tennessee, 2016-2022

**DOI:** 10.1017/ash.2025.414

**Published:** 2025-09-24

**Authors:** Daniel Muleta, Raquel Villegas, Srilakshmi Velrajan, Cherlly Bailey, Jackie Taylor, Dipen M Patel, Melphine Harriott, Michael Norris

**Affiliations:** 1 Tennessee Department of Health; 2 state of TN; 3 TN Department of Health; 4 TN Dept of Health; 5 Tennessee Department of Health; 6 TN Department of Health HAI/AR Program; 7 State of Tennessee Department of Health

## Abstract

**Background:** Carbapenem-resistant Enterobacterales (CRE) have become an increasing public health challenge in the United States over the past two decades. Carbapenemase-producing CREs (CP-CREs) significantly contribute to the spread of antimicrobial-resistant pathogens in healthcare settings. Tennessee has been conducting surveillance of CRE since 2011. As part of the Emerging Infections Program (EIP), the state has participated in population-based surveillance in Davidson and seven surrounding counties, collaborating with the Centers for Disease Control and Prevention (CDC) since 2014. **Methods:** The data collected through the Muti-site Gram-negative Surveillance Initiative (MuGSI) project, a collaboration between Tennessee and CDC as part of EIP, was used for this study. The analysis was performed on a subset of CRE isolates tested for carbapenemase production (CP) among all incident CRE cases collected from 2016 to 2022. Incident CRE cases are defined as the identification of carbapenem-resistant E. coli, Enterobacter cloacae complex, and Klebsiella species (K. aerogenes, K. oxytoca, K. pneumoniae, and K. variicola) from urine or normally sterile specimens (e.g., blood) from the residents of the surveillance area in a 30-day period. The mortality data was obtained from the Tennessee Vital Registry and merged with the surveillance data. Cox regression analysis was performed to evaluate if there is a difference in the 90-day survival rate based on the CP status of the pathogen, gender, age group, and the Charlson comorbidity index (CCI) score. Data analysis was done using SAS version 9.4. **Results:** There were 570 CRE cases reported during the study period (2016-2022). Of these, 406 were tested for carbapenemase production and 87 (21.4%) were positive for CP. There were 269 (66.3%) females and 137 (33.7%) males. Patients with higher Charlson comorbidity index score (> = 5) have significantly higher hazard ratios compared to those with low scores (HR 4.17; p-value) **Conclusion:** This study indicates that patients infected with CP-CRE, females, and those with high Charlson comorbidity index score have a significantly higher probability of dying within 90 days. These factors are worth considering when conducting a risk assessment of patients infected with drug-resistant gram-negative bacilli. The significantly increased risk of death among patients infected with CP-CRE highlights the need for timely carbapenemase testing and use of the test result for appropriate antimicrobial therapy and infection prevention.

**Figure:**
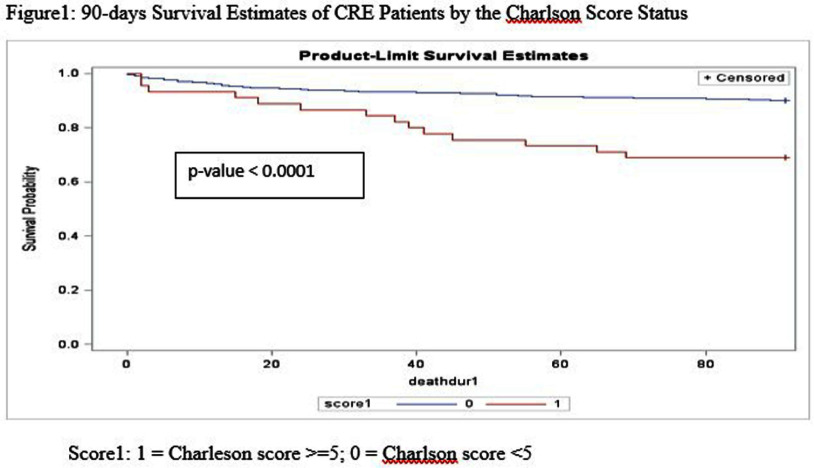

**Figure:**
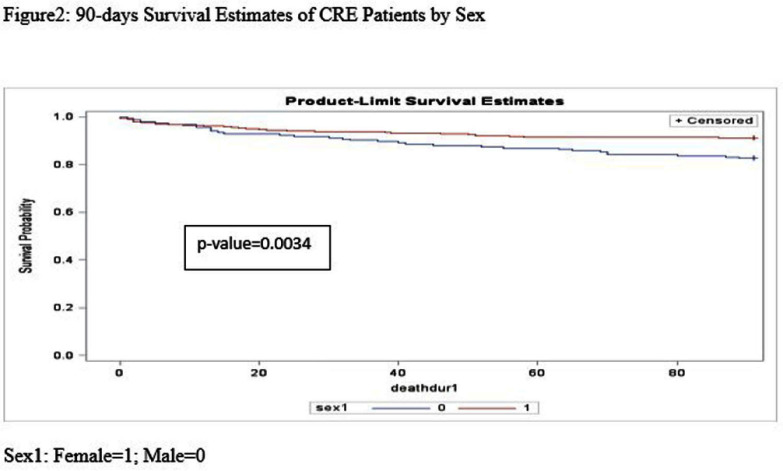

**Figure:**
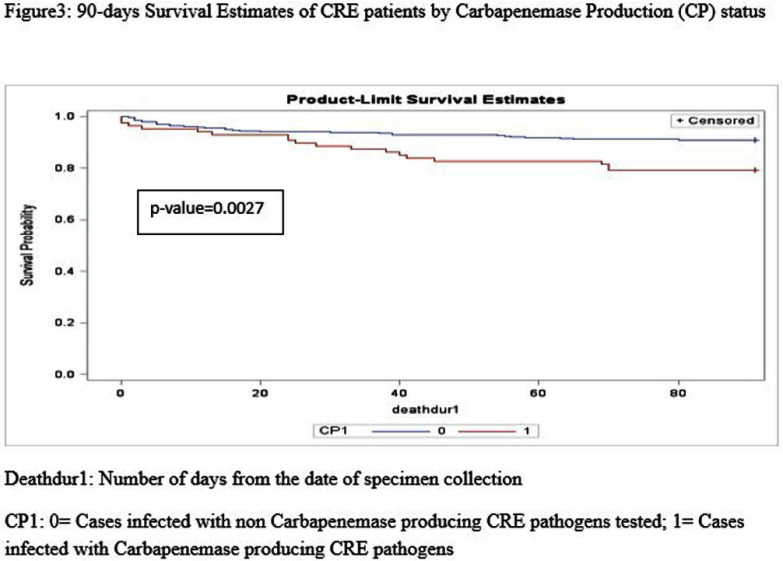

**Figure:**